# On the Origin of Life on Earth: The Nanozymes Hypothesis, and More

**DOI:** 10.34133/research.1025

**Published:** 2025-12-09

**Authors:** Yongdong Jin

**Affiliations:** Marshall Laboratory of Biomedical Engineering, Guangdong Key Laboratory of Biomedical Measurements and Ultrasound Imaging, School of Biomedical Engineering, Shenzhen University Medical School, Shenzhen University, Shenzhen 518060, P. R. China.

## Abstract

The origin of life (OoL) is a fundamental and long-standing scientific question. Although a variety of plausible hypotheses had been put forward, how life began on the prebiotic Earth from a pile of prehistoric inert chemicals (gases) is still a puzzle to us. Here, to unify the existing hypotheses to cover the entire scenarios, the author proposed the “nanozymes hypothesis” of the OoL on Earth, in which natural mineral nanozymes (MN-zymes) and their later upgraded organic/inorganic hybridized nanozymes played multiple key roles in the initial emergence of life molecules, especially in the manner of “inorganic photosynthesis” under primitive Earth conditions. Under the hypothesis framework, proteins, DNA, and RNA might emerged near-simultaneously, as a result of the diversity of nanozymes and catalyses, and multiple physical and chemical key roles of the MN-zymes. Besides nanozyme aspects, several fundamental and key issues on the topic are briefly discussed and several essential elements and conditions for the natural selection and survival of life molecules are proposed.

## Introduction

The emergence of the first biopolymers and their building blocks on the early Earth is considered a key moment in the origin of life (OoL), but how life began on the prebiotic Earth from a pile of prehistoric inert chemicals (gases) is still confusing, and the search for its truth is often even more so because the full scenarios are difficult to recreate. Over the past century, a variety of plausible OoL hypotheses have been proposed, mostly centered on (terrestrial or interstellar) chemical origin/evolution theories [1–3], but there are still a lot of controversy and incompleteness about these hypotheses because each of them builds upon one-sided empirical data and/or certain theoretical bases. For instance, numerous theories explaining the (terrestrial) chemical OoL, such as the Metabolism-first world (FeS world) [4], Zinc world [5], Thioester world [6], RNA world [7], Lipid world [8], and so on, had been proposed. However, each of them can only explain relatively narrow parts of whole scenarios, no convincing “one-piece” hypothesis so far can unify them, to provide complete and credible scenarios of how original life can arise from a bunch of inert chemicals on the planet.

Recently, nanoparticles (NPs) with enzyme-like characteristics (coined as nanozymes) [9,10]—a hot pursuit of current research—aroused broad research interest, among which (mineral) nanozymes (MN-zymes) are gradually considered to play a crucial role in the OoL [11,12]. After deeply conceiving the diverse roles of (artificial) nanozymes, the author has become increasingly convinced that the nanozymes hypothesis proposed in this article would be the optimal and the closest one to the truth. In the scenario of the nanozymes hypothesis, in addition to the participation of natural MN-zymes, some important physical and chemical processes of MN-zymes played a key role in creating the Earth’s first living matter, such as anti-ultraviolet (UV) irradiation, physical/chemical catalysis, self-assembly and self-organization, self-compartmentalization and pairing stabilization, and so on.

It is well known that biological enzymes played important roles in the evolution of existing organisms and living organisms today, but strangely, the possible similar roles of their initial inorganic analogs, MN-zymes of the Earth, in the creation of living materials and the birth of life on early Earth were completely overlooked. In fact, naturally formed NPs of minerals are plentiful on Earth. Annually, thousands of terragrams (Tg) (1 Tg = 10^12^ g) of mineral NPs on Earth move around in natural ecosystems; some of them exhibit intrinsic enzyme-like characteristics (termed MN-zymes), which are ubiquitous in the oceans, waters, atmosphere, and soils, and play critical roles in environmental biogeochemical cycles [13]. Moreover, recent new discoveries and conjecture about the roles of natural MN-zymes in the origin of early life on Earth, such as peptide formation in a volcanic SO_2_ environment with Hadean mineral covellite (CuS) (related to Peptide world) [14], hydrated spin-polarized electron generation that induces enantioselective prebiotic chemistry by UV (200 to 300 nm) irradiation of magnetite (Fe_3_O_4_) deposits (on Life’s Homochirality issue) [15], and critical roles of the surface of minerals and geochemical complexities on the OoL [12], have also re-aroused research interest in the field. Therefore, based on these and my long thinking on the fascinating topic and current explosion advances in artificial nanozymes, the author proposes herein the “nanozymes hypothesis” of the OoL (on Earth).

## Nanozymes Hypothesis on the OoL (and Its Evidence)

In the OoL hypothesis, primitive MN-zymes and their later upgraded organic small molecule hybridized nanozymes played a crucial role in the long history of the birth and evolution of life on Earth, especially in the early stage of the advent of life molecules and materials. They gradually catalyzed the generation of prehistoric small life molecules from nonliving matter, a bunch of prehistoric inert chemicals (gases), via complex chemical (and physical) processes in the main way of “inorganic photosynthesis” under primitive Earth conditions. It should be specified that, not entirely equivalent to today’s traditional nanozymes that are defined as nanomaterials (NMs) with enzyme-like activities that exhibit similar catalytic behaviors or kinetics to natural enzymes, in the context of the OoL, the catalytic reactions carried out by the nanozymes (MN-zymes) proposed herein are different from those of natural enzymes and even far inferior to those of today’s nanozymes, especially in their early stages as primitive MN-zymes.

In the big natural laboratory of the Earth, with the assistance of some key physical and chemical processes under the Earth’s harsh environment conditions (relatively for life, but some are in fact milder than today’s laboratory chemical reactions) at that time, they constantly iterated and renewed themselves, co-synthesized, and catalyzed various organic small molecules to evolve the early life forms on Earth. The emergence of life is therefore definitely a natural outcome of the interplay between solar radiation and multiple Earth condition on primordial Earth, rather than merely an accidental event, and among them, MN-zymes played critical roles, especially in the initial stage.

**Fig. 1.**
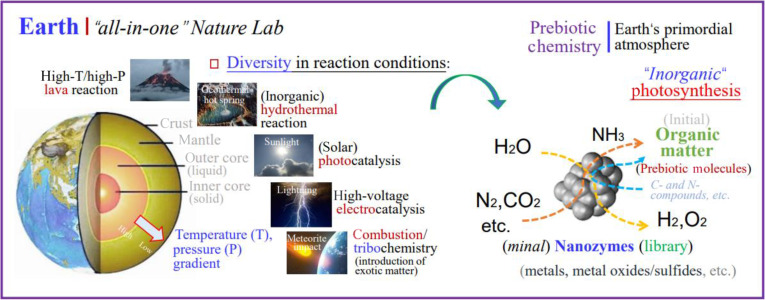
Schematic diagram depiction of the Earth as an “all-in-one” sustainable natural big Chem-Lab for producing prebiotic molecules for the OoL on Earth, under the primordial atmosphere and the MN-zymes (library) frame.

With the advancement of science and renewal of knowledge, I, maybe we, believed that the primordial Earth environments itself actually had the basic conditions to initiate and support the emergence of life. In fact, the Earth itself has the ability to gradually cultivate organic life and world from a completely all-inorganic primordial Earth environment and harsh conditions in the long history of the birth and evolution of life on Earth (akin to previous abiogenesis opinion) [5]. Within the framework of the OoL hypothesis, the Earth is a natural “all-in-one” big and sustainable chemistry laboratory. As schematically depicted in Fig. 1, the existence of natural pressure gradients and temperature gradients at different depths and locations (from mantle to crust) of the Earth, especially at the sites of active volcanos and geothermal hot springs, provides abundant conditions for high temperature/high pressure lava reaction and hydrothermal reaction to generate the initial MN-zymes (such as metals/noble metals, metal oxides, and sulfide NPs), the methods of which are actually being widely used for artificial nanozymes syntheses nowadays in laboratories worldwide. These primordial MN-zymes (library) had iterated slowly and self-renewed (upgraded), and some have even become part of living organisms over the long history of Earth’s evolution, and in turn resulted in the mineral evolution and mild change of the Earth’s environment [16,17], all of which provide improved conditions for better survival and evolution of prebiotic molecules and primordial life.

In fact, multiple natural phenomena on Earth can spontaneously produce MN-zymes in more effective ways than we thought, as recently discovered that NMs can be simply formed via weathering of natural minerals spontaneously in charged water microdroplets [18], or under UV irradiation, for example, forming ZnCd quantum dots (QDs) from a basic mixture of thiols (mercaptosuccinic acid) and Cd(II) and Zn(II) ions [11]. In addition, the natural sunlight conditions and lightning phenomena also provide sufficient photocatalytic and electrocatalytic reaction conditions for the upgraded mass production of primordial natural nanozymes and their later organic hybrid nanozymes, and the generation of rich prebiotic molecules on the Earth’s surface.

Table 1 presents a preliminary summary of the literature survey [9,11,19–26], showing possible reaction activities and prebiotic molecules generable on Earth by some main MN-zymes (earth-abundant NMs) with (protoenzyme-like) catalytic activity, such as magnetite (Fe_3_O_4_), FeS, and ZnS NPs. The initial catalytic chemical reactions of these primitive mineral NPs (MN-zymes) provided basic and necessary biogenic carbon, nitrogen, phosphorus, and oxygen (C/N/P/O) sources and cycles for the synthesis of prebiotic molecules on Earth. Notably, besides other catalytic roles of MN-zymes, the photocatalytic reduction of CO_2_ (one of the main components of the primordial atmosphere) and H_2_O into O_2_ and hydrocarbon molecules, which I refer to here as “inorganic photosynthesis” (i.e., prebiotic photoredox/photocatalysis) (with sunlight as an energy source to produce carbohydrates and O_2_ from water and CO_2_, the term similar to the known “artificial photosynthesis” in today’s catalysis and energy fields) [23], undoubtedly played a vital role in the long history of the primordial OoL on Earth. The so-called “inorganic photosynthesis”, akin to the organic photosynthesis of plants that emerged later on Earth (the process by which green plants turn CO_2_ and H_2_O into O_2_ and chemical energy using sunlight), would be the initial key step for primitive carbon-based life on Earth—though the reaction efficacy would be low as compared to the latter. The process creates an aerobic atmosphere and a readily usable carbon source to promote the birth and survival of primitive carbon-/oxygen-based life on the planet. The MN-zymes listed in Table 1 are classified into 2 stages, “stage I” and “stage II”, based on their chronological order, and catalytic capacity and diversity. In stage 1, the (organics-free) primitive MN-zymes generate and provide mainly and only the most fundamental O, C, N-based gases and small molecules, including initial primitive steps toward carbon fixation. In the hypothesis, primitive MN-zymes (stage I) and their later upgraded organic small molecule hybridized nanozymes (stage II) played a crucial role in the long history of the birth and evolution of life on Earth, for the early advent of life molecules and materials.

**Table 1.** A preliminary survey listing reaction activities and prebiotic molecules that can be generated on Earth by earth-abundant main MN-zymes (i.e., geologically plausible mineral catalysts), the results of which were inferred from the artificial/synthetic analogs/systems under simulated or similar conditions

Mineral nanozyme (Earth-abundant NMs)	Reaction activities and products (reported based on similar artificial one)	Reference
Iron-based NMs: magnetic (Fe_3_O_4_) NPs γ-Fe_2_O_3_, γ-FeOOH	Peroxidase (POD)-like activity, H_2_O_2_→O_2_ catalase-like activity, $2H_{2}O_{2}\overset{\text{Catalase}}{\to }2H_{2}O+O_{2}$	[9,19]
Cerium oxide-based NMs	Superoxide oxidase (SOD), catalase, or oxidase (inverse reactions) mimics; $2{O_{2}}^{•-}+2H^{+}\overset{\mathrm{SOD}}{\to }H_{2}O_{2}+O_{2}$	[9]
CuO, MnO, V_2_O_5_, Co_3_O_4_, Au and Pt NPs, etc.	Oxidase, POD, SOD, and/or catalase mimics	[9]
Nano-sized TiO_2_, etc.	Photocatalytic water-spitting for H_2_ production	[20]
TiO_2_, Fe_3_O_4_, Fe_2_O_3_, MnO_x_, MoS_2_, etc.	Electrocatalytic ambient NH_3_ synthesis from N_2_ and H_2_O (enough nitrogen sources for nitrogenous compounds)	[21]
Stage I: pristine mineral NP (nanozymes) catalysis (usually with low efficiency/poor selectivity		
Artificial nanozymes based on Cu, Fe/Ni, Cu/Zn, NiO, g-C_3_N_4_, etc.	With β-glucoronidase and pan-enzymatic activity	[22]
Solar-generated ZnCd QDs [from Zn(II)/Cd(II) in the presence of thiol capping agents]	Xanthine oxidase (XO)-like activity	[11]
Semiconductor metal oxide sulfides, nitride, and phosphide NPs, such as TiO_2_, CuO, FeS, ZnS, and their hybrids with Pt/Ag/Au, etc.	Photocatalytic reduction of CO_2_ into short-chain carbohydrates (CH_4_, CH_3_OH, HCOOH, C_2_H_6_, etc.)	[23]
Metal NPs (Pd, Au, Ag, Pt, Ni, or bimetals, etc.)	Photocatalytic water splitting and photo-hydrogenation of alkenes, alkynes, and CO_2_), hydrogenation of unsaturated substrates, and oxidation; oxidation of hydrocarbons and alcohols, etc. (especially for PdNPs: catalytic formation of C–C and C–N bonds)	[24,25]
Fe_3_O_4_-based NPs, etc.	Homocoupling (C–C) and heterocoupling (C–O, C–S)	[26]
Stage II: self-evolved/upgraded nanozymes catalysis (conjugated with organics, with improved catalytic efficiency and diversity, creating conditions for the birth of early life)		

NMs, nanomaterials; NPs, nanoparticles

The primitive inorganic MN-zymes are thought to catalyze the synthesis of the initial (bio)molecules and can polymerize the formation of longer (bio)molecules (oligomers) on their surfaces [27], which are very important for the formation of primitive biomolecules. Currently, various artificial nanozymes made of thousands of different NMs have been developed since the first report of Fe_3_O_4_ NPs with peroxidase-like activity [19], with performance of single-atom nanozymes even comparable to natural enzymes [28]. Therefore, there is no doubt that the rapid advances in this field will help us gradually and comprehensively figure out the complete biomolecular evolution scenarios of the hypothesis. The author assumes here and believes that in the world of the nanozymes hypothesis, proteins, DNA, and RNA could emerge near-simultaneously, as a result of the diversity of nanozymes catalysis. This will answer another important puzzle of the OoL—“Which came first, DNA or proteins?”—and helps to explain RNA world hypothesis. Fortunately, so far, diverse (mineral) nanozymes have been experimentally found to play key roles in almost every major scenario of the OoL, such as the tricky issues of the origin of Chirality and the RNA world. In addition to the capability of MN-zymes to catalyze the primitive organic synthesis (such as carbon fixation) in the primordial environment [29,30], recently we know that the chiral molecule selection can realize on the chiral surface of natural minerals like the calcite [31] or by hydrated spin-polarized electron generation by UV irradiation (which is strong on primitive Earth) of magnetite (Fe_3_O_4_) deposits [15]. Moreover, except for the synthesis and concentration of small molecules, the polymerization of the peptides and RNA molecules can also be catalyzed by clay minerals, like layered double hydroxide minerals [32,33]. Box 1 summarizes the abovementioned several key elements of the hypothesis for better understanding of readers.

Box 1. A few key points in the hypothesis

• MN-zymes and their later upgraded organic small molecules hybridized played a crucial role in the long history of the birth and evolution of life on Earth;
• Natural conditions on primitive Earth can spontaneously produce MN-zymes in more effective ways than we thought;
• Photocatalytic reduction of CO_2_ into hydrocarbon molecules, i.e., “inorganic photosynthesis”, played a vital role in the long history of the primordial OoL on Earth;
• Mutual transformation and self-adaption process occur between the natural MN-zymes and prebiotic molecules and primordial organisms on Earth;
• Proteins, DNA, and RNA probably can form spontaneously at the same time due to the diversity of nanozymes and catalyses, within the framework of the hypothesis.

## Multiple Critical Roles of Natural MN-zymes in the OoL

**Fig. 2.**
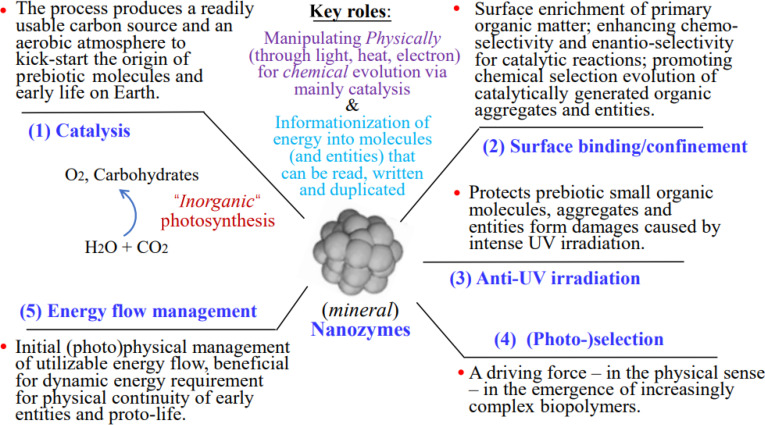
Multiple critical roles of natural MN-zymes in the OoL on Earth.

Figure 2 highlights the multiple critical physical and chemical roles that natural MN-zymes may have played in the OoL on Earth, including (a) catalysis, (b) surface binding/confinement, (c) anti-UV irradiation, (d) (photo-)selection, and (e) energy flow management. By playing these multiple roles, MN-zymes not only manipulate themselves physically via light, heat, and electricity, for initiating chemical evolution of prebiotic organic molecules, but also enable informationization of energy into molecules (and entities) that can be read, written, and duplicated, all of which are necessary for the origin of living systems.

As depicted in Fig. 2, besides the main and diverse roles of catalysis (a) to produce a readily usable prebiotic organic molecules (including C/N/O source) and an aerobic atmosphere—to kick-start the origin of prebiotic molecules and early life on Earth in which the “inorganic photosynthesis” played a critical role—natural MN-zymes also played important roles in other important scenarios and key events in the OoL. For instance, MN-zymes, especially AuNP-based nanozymes and their surfaces, provide ideal binding and reaction sites/platforms via surface binding/confinement effect (b), for the enrichment of primary organic matter [34], enhancing (surface-confinement induced) chemo-selectivity and enantio-selectivity for catalytic reactions [for instance, based on the chiral-induced spin selectivity (CISS) effect of magnetite deposits in shallow prebiotic lakes by solar UV irradiation] [15], and promoting chiral selection [31] along with promoting chemical selection evolution of catalytically generated organic aggregates and entities [34]. Life molecules as we know is homochiral, but its origin of biological homochirality on early Earth remains unclear. Since iron-containing minerals, e.g., ferrihydrite, hematite, and magnetite, and other minerals are ubiquitous in Earth systems [13], these very recent findings based on natural (nano)minerals favor the nanozymes hypothesis and will help to uncover the mystery of homochirality origin. Notably, molecules adsorbed on the surface of nanozymes such as AuNPs are able to diffuse along the surface, interact with each other, and form aggregates, which is a precondition for the abiogenic emergence of increasingly complex organic structures, and the genuine fight for survival and evolution could start on it as each replicator became a potential prey for others.

Although the very lack of oxygen in the primordial atmosphere favored light-driven prebiotic syntheses for MN-zymes (e.g., the catalytic transition of formamide into nucleobases occurred on TiO_2_ surfaces powered by UV) [35], the solar light reaching Earth without the ozone shield contained a UV component that was 10 to 1,000 times stronger than it is today [5]. The heating and reactive oxygen species (ROS) generated by strong UV radiation were therefore one of major environmental dilemmas for the emergence of early life on Earth then. But such problem/paradox can be mitigated or resolved due to the anti-UV irradiation ability (c) of MN-zymes, especially for ZnS in the “Zn world” hypothesis scenario [which utilizes solar UV light as an energy source both for prebiotic syntheses and for (photo)selection, and in accord with the RNA world hypothesis and reconcile and combine the “metabolism first” and “replication first” concepts] [5]. This is because MN-zymes can protect prebiotic molecules, especially the later formed/evolved nucleobases, DNA and RNA molecules from photo-dissociation or photo-damage, by mainly taking advantage of radiation energy from the adsorbed photoactive compounds (the bound polypeptides and sugar-phosphate backbones), and can simultaneously deliver UV energy for polymerization reactions to connect nucleotides and/or polypeptides together [5]. Furthermore, the scenario of “Zn world” also suggests that porous ZnS formations of hydrothermal/volcanic origin were involved in and enabled the (photo)selection (d) of the first polypeptides and RNA-like polymers (the first replicating entities) [5], the role of which is considered a critical (physical) driving force for the advent of increasingly complex biopolymers, including first replicators and enzymes. To our relief, the potential roles of nanozymes, especially their enzyme-like activities, in the OoL have aroused increasing attention and research interest in recent years, and existing studies have also preliminarily illustrated the positive contribution of diverse functions of nanozymes in different stages of chemical evolution [36]. However, many critical issues need to be further clarified and experimentally proved in the future.

It is also worth noting that, to facilitate the origin and evolution of life, living organisms need to build a dissipative structure far from equilibrium state in order to survive adverse environments. Since living bodies need the exchange of both matter and energy with the outside world to maintain the dissipative structure, energy flow management (e) by MN-zymes would also play vital roles for initial (photo)physical management of utilizable energy and flow (such as solar UV light, lightning, and hydrotherm) to keep their own entropy at a lower level than the environment while consuming materials and energy.

## A Possible Chemical Evolution Route of OoL on Early Earth Based on MN-zymes

**Fig. 3.**
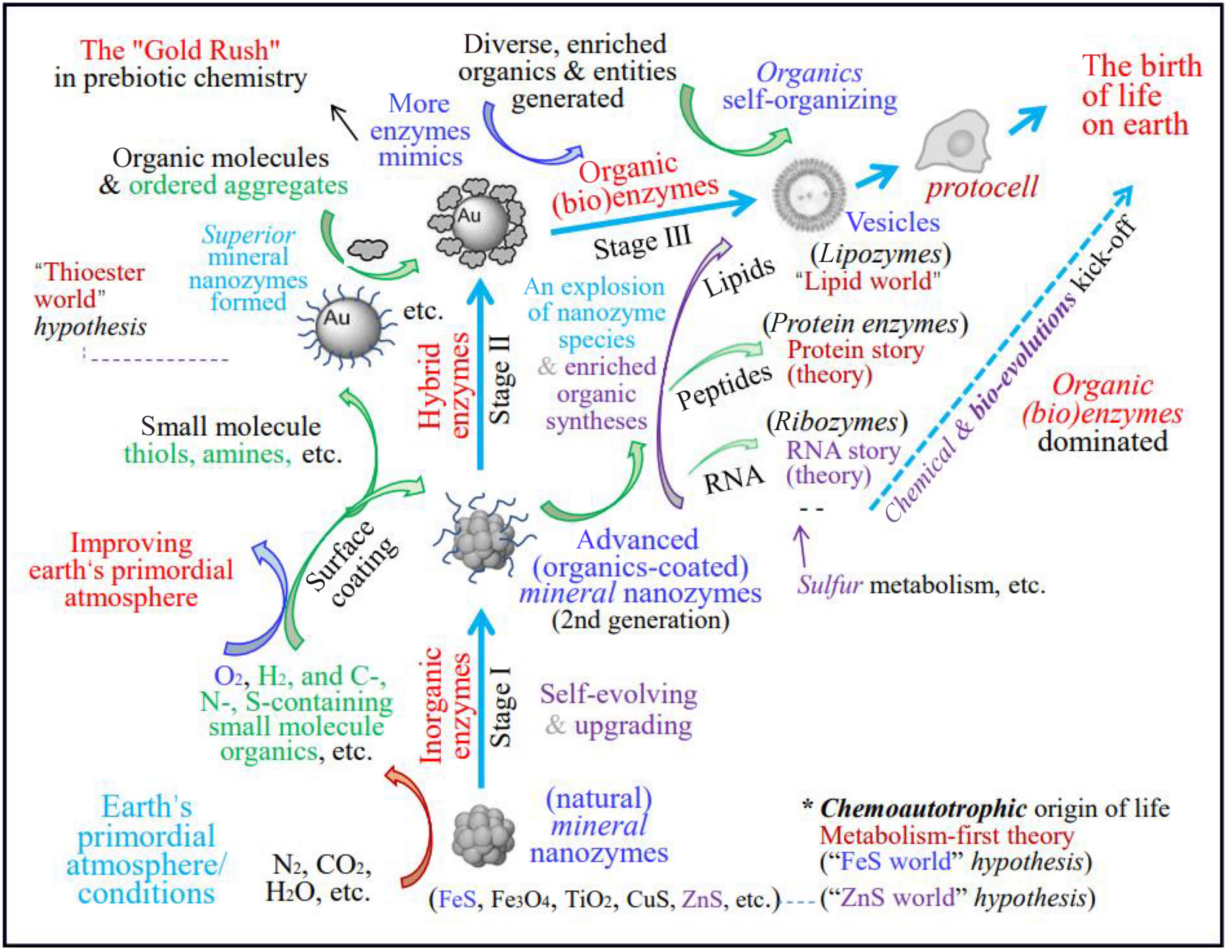
Presumed chemical origin route and evolutionary family tree of MN-zymes in the OoL on Earth.

Figure 3 depicts the presumed chemical origin route of prebiotic life on Earth under the framework of the nanozymes hypothesis. Prior to the emergence of prebiotic molecules, natural MN-zymes (ZnS, FeS, etc.) and their later slowly self-upgraded nanozymes—more functional inorganic/organic hybrid MN-zymes (with surface modified by small organic molecules that naturally produced)—catalyzed the generation of prehistoric small life molecules, from a bunch of prehistoric inert chemicals (gases), under mainly the “ZnS/FeS world” hypothesis scenarios, through the main way of “inorganic photosynthesis” under primitive Earth conditions. The processes provided initial necessary biogenic carbon, nitrogen, phosphorus, and oxygen (C/N/P/O) sources and cycles to kick off the OoL on Earth. Meanwhile, the continuously generated oxygen by “inorganic photosynthesis” helped to improve primordial atmosphere gradually, benefiting the emergence of life on Earth. Over the long history of the natural evolution, these primordial MN-zymes (library) had iterated slowly and self-renewed (upgraded), and some later even became part of living organisms. The initial inorganic MN-zymes catalyze the syntheses of the initial small (bio)molecules and can polymerize the formation of longer (bio)molecules (oligomers) on their surfaces [27], while in later stage, proteins, DNA, and RNA precursors or analogs can be spontaneously formed almost simultaneously at a certain stage (the Big Bang of molecular diversity), as a result of catalytic diversity caused by increasingly abundant and iterated inorganic/organic hybrid nanozymes. Generally, it is assumed that in the earliest stages of life, inorganic MN-zymes, including mineral clays, played a crucial role in concentrating small molecules and catalyzing their chemical transformation (i.e., surface metabolism) [37], through surface attachment and surface-confined catalytic reactions, and would at some point lead to the formation of big biomolecules (lipids, peptides, and RNA) that are capable of structuring themselves and taking up catalytic roles independently from the surface.

It is worth noting that, among all working MN-zymes, the author believes that monolayer-protected gold NPs (AuNPs) as superior MN-zymes, though currently unthought of and severely overlooked, participated in and played key roles in family tree of MN-zymes in the OoL on Earth—which I refer to as the “Au world” here. In fact, AuNP, which is today more often regarded as an artificial nanozyme, was a geologically plausible MN-zyme that exists naturally. Recently, more and more scholars have come to realize that natural AuNPs are an important component of gold under various geological conditions of the Earth, such as hydrothermal fluids, black chimneys on the seabed, hydrothermal gold deposits, and supergenic gold deposits; so far, natural AuNPs have been discovered in gold deposits such as Carlin-type gold deposits, orogenic gold deposits, skarn-type deposits, weathering crust gold deposits, and sedimentary rock-type gold deposits [38,39]. Although it may be hard for free AuNPs to exist in the original soup (since they can hardly stably exist without surface coating of organic molecules), AuNPs may survive on the surface of some deposits and can exist primarily in the (thiols/amines) monolayer-protected forms [34] after small molecules like thiols and amines are produced (by other MN-zymes) and enriched at some sites. As AuNP-based MN-zymes (family) have sufficient stability, moderate organic molecule surface binding/dissociation capacity, and rich and versatile surface chemistry, as well as their ability to display primitive adaptive behavior and install rudimentary signaling pathways [34], we can boldly assume that a species explosion of MN-zymes and organic molecule syntheses occurred at some time on Earth, although herein the evidences and scenarios from artificial AuNP nanozymes may be overextended to prebiotic conditions.

Moreover, the single chirality of biological molecules is a signature of life, but how it emerged is also one of the greatest mysteries of the OoL on Earth. The emergence of AuNP-based organic/inorganic hybrid nanozymes may also played key roles in the process as scientists recently discovered a group of sulfur-based molecules that may have existed in the early Earth that can easily link individual amino acids to amino acid precursors (called aminonitrile) to form dipeptides, and importantly, they exhibit monochiral selectivity [40]. During this “Golden” period, represented by AuNP nanozymes, the Earth entered mainly the hypothesis scenarios of “lipid, protein, and DNA and RNA worlds”, promoting the formation and birth of diverse key biomolecules and vesicle-like protocells, including the emergence of RNA molecules, ribozymes, and lipozymes, which act as initial prebiotic organic enzymes [8], to kick off chemical and biological evolutions. Although gold is a precious metal with relatively low natural abundance in geological environments, and one may doubt its contribution to prebiotic chemistry, time may solve this problem and provide an answer in the long history of the OoL. Just as from the perspective of the universe, the OoL on Earth itself is an extremely low-probability event. Note that under the framework of various previous OoL hypotheses, DNA cannot be synthesized in living organisms without the catalytic action of proteins as enzymes, while without DNA as the guidance of genetic information, amino acids also cannot be combined into protein molecules with specific functions. With the emergence and support of more and more experimental evidence in the future, this paradox will be figured out gradually under the framework of the nanozymes hypothesis.

How the MN-zymes later transitioned to biological enzymes is, therefore, another key open question encountered within the framework of the OoL hypothesis. Although the exact causes require more research and time to reveal and prove, the author believed that natural selection played decisive roles in it. In the long process of life evolution, especially in the later stage of it, the fast, highly efficient, and self-adaptive biological evolution gradually replaced the initial slow and low-efficiency physical/chemical evolution and took the dominant position, because biological enzymes are more efficient and renewable than their parent MN-zymes in the life world, resulting in the smooth “handover” of MN-zymes systems to biological enzymes eventually. Although the vast majority of MN-zymes have withdrawn from current life systems, some remnants and traces of them can still be found in some animals and plants, and some have even become part of living organisms to continue to influence the mineral evolution and mild change of the Earth’s environment [16,17], as aforementioned.

## More on the Hypothesis (Beyond MN-zymes)

In addition to the nanozyme aspect of this hypothesis, there are several fundamental and key issues about the OoL on Earth that need to be briefly discussed and further addressed.

(a) The material selection basis and essential elements for the OoL on Earth. We know that living organisms are dissipative structures far from equilibrium; simplifying many possible compositional configurations to as few specific compositions as possible has therefore been considered as an important step in the invent of life [8], where the formation and diversity of vesicular structures should play the key roles. In fact, amphiphilic molecules were likely relatively abundant in the prebiotic environment under the nanozymes hypothesis framework, as virtually any molecule that contains a long enough hydrocarbon moiety and a polar group is an amphiphile, and vesicular structures have been shown to have the capacity to capture light energy by incorporation of pigment molecules that partition into the bilayer, or redox energy by mediating electron transport reactions across the membrane to produce electrochemical potentials, to power a life [8]. To better understand basic principles of the OoL from a physical and chemical perspective, the author proposed herein further 4 essential elements and conditions for the natural selection and survival of life molecules regarding the OoL on Earth, as shown in Box 2, namely, wet–dry cycling and amphiphilism, self-assembly and self-organization, catalytic and protoenzyme activity, and pairing symbiosis and stabilization.

Box 2. Four essentials and conditions for the OoL on Earth

• Wet-dry cycling and amphiphilism—for molecule enrichment and vesiculation;
• Self-assembly/self-organization—for survival and compartmentalization of living organisms;
• Catalytic and protoenzyme activity—for biological functionality, activity, and evolution;
• Pairing symbiosis and stabilization—for stability and heredity of living entities

(b) The importance of the Earth’s superficial structure and unique water properties/environment. Water is believed to be essential for the OoL, at least on Earth. But a major doubt about it is the Water paradox [41], which states that although water is key to the formation of life, it can also destroy the core ingredients of life, because vesicles or protocells are hard to be formed in diluted amounts of water and the building blocks of life such as proteins and nucleic acids like DNA and RNA are prone to break down in water. Today, cells solve this problem by limiting the free movement of water within them, but it was a tricky problem at the very beginning of primitive life. That is why researchers abandoned the hypothesis that life originated in the ocean (hydrothermal vent scenarios, too much water there) and instead began to focus on terrestrial environments, particularly those that alternated between wet and dry (such as small ponds). Although the competing hypotheses (hydrothermal vent scenarios versus terrestrial ponds) have long conflicted with each other due to the existing paradox of water, within the framework of the nanozymes hypothesis, the conflict can be naturally resolved and coexist reasonably. MN-zymes played different key roles and functions in the 2 distinct geological scenarios: They were mainly produced at the hydrothermal vents, but worked as catalysts to initiate the syntheses of prebiotic life molecules mainly at the rim of wet–dry cycling terrestrial ponds where they survived.

This opinion may have been confirmed as scientists recently discovered that amino acids can spontaneously link up to form protein-like chains when completely dry [42], and such reactions are easier to achieve for the 20 amino acids found in today’s natural proteins. Importantly, researchers also found that by mixing lipids and nucleotides with water and letting them go through the wet and dry cycle, RNA-like polymers can be synthesized non-enzymatically in lipid (vesicle) environments from mononucleotides under simulated prebiotic conditions—an important reaction for the invent of life but cannot be done in water without these extra helps [43]. Moreover, and equally important, during each wet cycle, weaker molecules, or those that cannot combine with others to protect themselves, will be destroyed, thus resulting in natural selection of life molecules, and providing a physical/chemical evolutionary path. In the wet and dry cycle area, activation chemistry may also drive the emergence of functionalized protocells, which has been preliminarily demonstrated in recent experiments [44].

In addition, we know that synthesis of the first RNAs represents one of the cornerstones of the emergence of life, and recent studies demonstrated powerful scenarios of prebiotic synthesis of cyclic nucleotides in aqueous and formamide environments. But their thermodynamic stability is a major concern as it is a decisive factor determining their accumulation in a prebiotic pool. A recent study based on computational thermodynamics pointed to the important role of environments (in formamide, or mixed aqueous solvents) to stabilize cyclic nucleotides, taking 2′,3′ and 3′,5′ isomer cyclic nucleotides as examples [45]; it offers a solvent medium-specific perspective on the stability of prebiotic synthesized cyclic nucleotides.

**Fig. 4.**
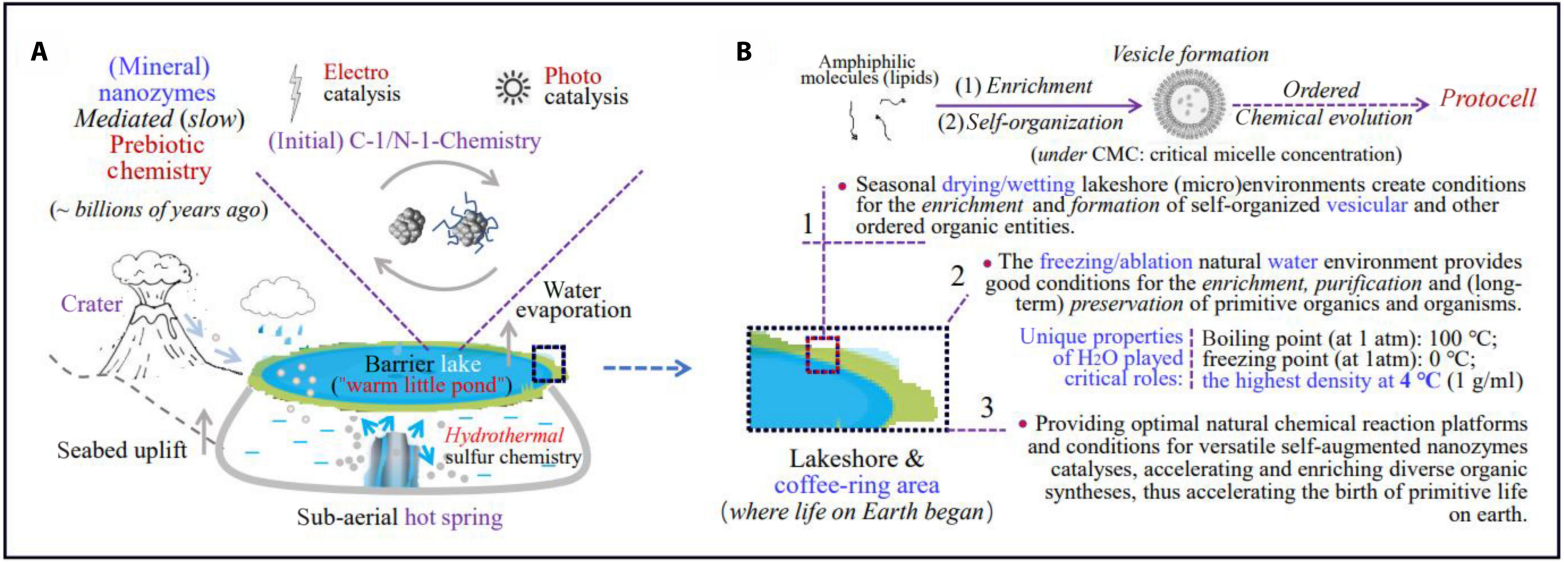
Schematic of macroscopic and microscopic enrichments and ordered organization of MN-zymes and organics for the OoL matters on Earth. (A) Schematic of macroscopic enrichment of MN-zymes and prebiotic organics driven by early Earth environment. (B) Schematic of microscopic, physically and chemically driven enrichment, and ordered organization of organics and the prebiotic origin of protocells.

Figure 4 shows the conceived scenarios of the most likely sites that primitive life originated on Earth. The terrestrial “warm little pond” near crater rich in underground hot springs would be preferred, as it provides necessary conditions for the production and enrichment of MN-zymes and prebiotic organics. Excitingly, recent supportive study revealed that a subterranean network of interconnected rock fissures (something akin to a distillation enrichment system in a chemistry laboratory) can accumulate and enrich prebiotic compounds driven by heat flows with efficiency up to 3 orders of magnitude [46], which facilitated materials reserve/enrichment and reaction conditions of organic primitive small molecules for the emergence of early life.

Compartmentalization is important and necessary for cells to survive. The lakeshore, particularly the “coffee ring area” where alternated wet-and-dry cycle frequently occurs, would be the best warmbed for the origin of protocell and primitive life. The surface soil and rock (micro) environment of the seasonal drying/wetting cycled lakeshore and especially freeze-drying/ablation atmospheric conditions—in fact a commonly used method nowadays for preparing vesicles in laboratories—provided natural conditions for the enrichment/selection of organics, and formation and preservation/survival of self-organized vesicular and other ordered organic entities. The opinion is supported by recent finding that initiating acid-catalyzed polymerization at the surface of water films or droplets, which is the reactive phase during a wet–dry cycle in freshwater hot springs associated with subaerial volcanic landmasses, causes polymerization of nucleic acid monomers into long chains [47].

Undoubtedly, the unique physicochemical properties of H_2_O molecules played a key role in this process. The freezing point (0 °C, at 1 atm) and boiling point (100 °C) of water and especially its abnormal tendency of the density variation with temperature (the highest value at 4 °C, rather than 0 °C) make water an ideal life-nurturing medium/carrier for life in the liquid state, while a natural protective barrier for living matter is in the ice state. Because of this unique property, particularly its ability to form ice layer on the surface to act as an insulating layer or container, the fragile bio-organics/bio-molecules and early life inside it will be isolated and less destroyed, allowing them to survive against harsh cold and heat conditions of the early Earth.

Notably, the gelation from purely inorganic species could be another key geoscience factor in the OoL on Earth [48]; it may help explain the emergence of the first cells on Earth, as the gel-like environment may offer distinct advantages for the assembly of the first cell(s) and in fact the cytoplasm in cell is a gel in nature. A recent study showed that all-inorganic, mineral gels can be easily obtained using only water and the (natural) inorganic salts of (NH_4_)_6_Mo_7_O_24_^.^4H_2_O and FeCl_3_^.^6H_2_O in a fully biocompatible setting [49]—and often with clay as a hotbed, which can enhance transcription and translation for early life evolution [50]. Additionally, such inorganic gels have their potential to promote cell division and fusion [49], while its relatively stiff and stable framework will be less prone to swelling and burning and more resistant against photo-damage and thermal degradation (which was harsh in the early Earth). Last but not least, Earth’s natural rain may also play a crucial role in the emergence of primitive cells. Scientists recently demonstrated how rain helped primitive cells, made of tiny droplets of complex molecules such as proteins, lipids, and RNA (called “coacervate droplets”, long seen as a candidate for the first primitive cells), to form reticular walls 3.8 billion years ago, a key step in the evolution from tiny droplets of RNA to bacteria, plants, animals, and humans [51]. All of these will help to shed light on the mysteries of pre-cell life evolution in the prebiotic age.

## More Related Recent Studies Support the Hypothesis

### On the natural synthesis of life molecules

Recent study showed that the catalytic behaviors of FeS (both pure and doped with Ti, Mn, Ni, and Co, all of which were common in hot spring environments) are capable of reduction of CO_2_ to methanol driven by H_2_ under simulated hot spring vapor-zone conditions, and even stronger with UV-enhanced light (200 to 600 nm) and UV-visible light (300 to 720 nm), especially in the case of Mn-doped FeS (at 120 °C) [52]. The finding provides new insights into prebiotic carbon fixation mechanisms and solid support of the OoL in terrestrial hot spring environments.

The recently discovered prebiotic synthesis-mimic chemical reactions helped us to imagine the first chemical scenarios for the emergence of life on Earth. In 2022, scientists discovered a new OoL chemical reaction (via α-keto acids, not aldehydes) [1], by using cyanide (CN-), NH_3_, and CO_2_ (key component in the reaction)—all of which are thought to have been common on the early Earth, to form amino acids and nucleic acids (the building blocks of proteins and DNA) in the absence of enzymes. We know that in today’s cells, amino acids are made by precursor α-keto acids, using nitrogen and protein enzymes; this reaction is quite like what happens in cells today, except that it is driven by cyanide rather than proteins. Importantly, researchers also found that a by-product of the same reaction is orotate, a precursor to the nucleotides that make up DNA and RNA. This suggests that, under the right conditions, the same primordial soup could produce more vital molecules needed for the key ingredients of life, strongly supporting the terrestrial chemical origin hypothesis of life on Earth, and provides new perspectives for chemical origin of prebiotic systems [3]. Indeed, such work is astonishing as key life intermediates, such as α-keto acids, can be produced from the prebiotic chemical soup in the absence of enzymes. Undoubtedly, such reactions will further benefit from the MN-zymes, which also contributes to the diversity of generated prebiotic molecules for the OoL on Earth.

In 2023, researchers synthesized a 13-amino acid peptide, which forms a di-nickel cluster capable of robustly producing molecular H_2_ from protons and structurally analogous to the Ni–Ni cluster in acetyl-coenzyme A (CoA) synthase and the Ni–Fe cluster in [NiFe] hydrogenase, 2 ancient, extant proteins central to metabolism [53]. This implies that modern enzymes may likely evolved from simple peptide precursors on early Earth, since Ni was an abundant metal in the early oceans. Therefore, we can imagine that in the basic evolutionary path of the (en)zyme systems in the OoL process, apart from the crucial roles of primitive MN-zymes (stage I) in generating initial small organic substances from prebiotic soup, the later self-upgraded organic matters hybridized MN-zymes (stage II) and the contemporaneously self-formed catalytic prebiotic biomolecules (like peptide precursors) or their metal ion clusters (acting as initial proto-enzymes) played vital roles to accelerate the OoL process. Ultimately, with the birth of life, it leads to the evolution of the (en)zyme systems into and replaced by more effective and self-replicating and regenerative biological enzymes. Moreover, since proton-coupled electron transfer (PCET) is a basis for energy conversion and storage in chemistry and biology [54], providing a sustainable cycle for the biogenic elements of C, N, and P, and plays critical roles in modern photochemical and electrochemical organic syntheses [55], the author believes that PCET was prevalent in prehistoric life and played key roles for the interconversion of important small molecules such as O_2_/H_2_O, N_2_/NH_3_, and CO_2_/alkanes.

In fact, nature did in ways better than we think. Very recently, researchers found that spraying of water microdroplets (a common natural phenomenon on Earth) induces an electrical discharge and causes luminescent emission (microlightning), which results in chemical reactions in surrounding gas, and prebiotic organic molecules containing C–N bonds, such as cyanide, glycine, and uracil, can be synthesized purely naturally by spraying water droplets into a gas mixture containing N_2_, CH_4_, CO_2_, and NH_3_ at room temperature [56]. Nature has its own set of rules and ways for the de novo synthesis of life (i.e., constructing a new form of life based on completely synthetic components) [57], and we should also keep in mind that under non-equilibrium conditions, the overall catalytic performance (of the nanozyme systems and vesicular systems) will be enhanced [58].

Also notably, CoA is vital to all life on Earth, as its functional subunit, pantetheine, is crucial in many origin-of-life scenarios. In 2024, researchers succeeded in selective and high-yield prebiotic syntheses of pantetheine in water at room temperature by forming amino nitrile molecules from hydrogen cyanide (thought to be abundant on early Earth), which may answer the question of how pantetheine emerged on the early Earth [59]. This study challenges the view that life could not have originated in large amounts of water (such as sea) as water is too destructive for life; it also helps to answer or figure out how pantetheine helped the first protein and RNA molecules react chemically to form the first living things—which would strongly support our assumption that protein, DNA, and RNA could emerged near-simultaneously on the early Earth.

### Molecular cooperation and co-evolution at the dawn of life

In nature, animals huddle together for resisting cold, and similarly will do for prebiotic molecules (for better survival), in either physical or chemical manners. Nowadays, we know that interactions between proteins and RNA are the cornerstone of many important biological processes from transcription and translation to gene regulation, but little is known about the ancient origin of said interactions. Researchers recently hypothesized that peptide amyloids (protein-like aggregates) played a vital role in the emergence of life, as their repetitive structure renders itself to building interfaces with other polymers through avidity, and found that short RNA with a minimum length of 3 nucleotides can bind to peptide amyloids in a sequence-dependent manner [60]. The sequence-specific RNA–peptide interactions of the kind may offer a way to understand the big mystery of the origin of the genetic code.

Of all plausible OoL theories, the RNA world concept predicts that life evolved from more and more complex self-replicating RNA molecules. But the question of how this RNA world then moved on to the next stage is one of the most mysterious chicken-and-egg-like conundrums in evolution, as in later stages proteins mainly serve as catalysts for life, while the primary function of RNA is information storage. In 2022, chemists may have solved this key puzzle by demonstrating that RNA molecules are able to link short chains of amino acids together [61]. The study showed that noncanonical RNA bases can establish peptide synthesis directly on RNA, creating peptide-decorated complex RNA chimeric molecules, which implies the early existence of an RNA–peptide world, and from which ribosomal peptide synthesis may have emerged. The discovery would fundamentally shift to support the “RNA–protein world” and of course the nanozymes hypothesis proposed herein because the origins of RNA and proteins are linked rather than independent. Therefore, it is plausible that partially overlapping emergence trajectories for peptides, ribonucleotides, and proto-DNA analogs occurred, supported by recent demonstrations of RNA–peptide synergy and prebiotic phosphorylation pathways; however, definitive convergence timelines remain unresolved.

### More on the physical aspects of the OoL

Unlike the well-evolved DNA/RNA systems adopted today, the earliest lifeforms probably adopted a reduced set of codon sequences that were gradually completed over time, driven by physical, chemical, and combinational constraints (under the assistance of MN-zymes). But how it developed is a notoriously difficult question in science. Except for its important role in prebiotic chemistry, UV radiation has recently been considered a selection pressure for the evolution of early codon sequences. By quantifying the UV susceptibility from large pools of DNA protogenomes and testing the timing of evolutionary incorporation of codon sequences, researchers have recently shown how UV light could have played a key role in the evolution of the early genetic code of a DNA-based genome [62].

Besides, lightning may have contributed more to the birth of primitive life matter than we can imagine, but its role has been underestimated except for the well-known Miller experiment. Recently, researchers have shown through a re-run of simulated lightning experiments that lightning-induced electrochemistry is a possible way to activate an inert atmosphere and accumulate active carbon and nitrogen molecules (sources) necessary for pre-life synthesis [63], and a recent study has shown that in the presence of appropriate (nanozyme) catalysts, CO_2_ molecules can be converted to oxalic acid electrochemically and subsequently coupled with small N, S, and F molecules to convert to amino acids by electrochemical upgrading [64]. Therefore, lightning-induced plasma electrochemistry at gas/liquid/solid phases on the early Earth, with the aid of MN-zymes, could have generated high concentrations of N-/C-containing feedstocks locally and produced a range of reagents globally that were crucial for the emergence of life.

Last but not least, besides nano-size-and-scale effects, we should note that physical/chemical interfaces and Boundarics played crucial roles in the OoL on Earth, the importance the latter (Boundarics) also be rethought and recognized in biomedicine recently [65]. Furthermore, besides the established prebiotic surface catalysis (FeS/Fe clays, montmorillonite, ZnS, etc.), we should not overlook the geochemistry of impact craters, especially the adenosine phosphorylation assisted by meteorites under proton irradiation conditions [66], which could solve the major hurdle of the abiotic phosphorylation of nucleosides in OoL studies, providing an alternative explanation for the “Phosphate problem” of OoL on Earth by using reduced phosphorus sources like pyrophosphite or amidophosphite. Moreover, meteorite minerals under irradiation promote formation of adenosine monophosphate (AMP)/adenosine diphosphate (ADP)/adenosine triphosphate (ATP)-like species [66]; there is no doubt that the meteorite-assisted phosphorylation and impact craters as photochemical reactors contributed to and functioned as the energy flow management role of the MN-zyme systems for the OoL on Earth.

We should also note the possible important role of Fe clays and iron meteorites on prebiotic synthesis at impact craters, as a recent study showed that iron-rich smectites formed by reprocessing of basalts due to the residual post-impact heat could catalyze the synthesis and accumulation of important prebiotic building blocks such as nucleobases, amino acids, and urea [67]. This might support the claim that proteins, DNA, and RNA might emerged near-simultaneously, and implies that such matters and events may have emerged along partially overlapping trajectories enabled by diverse mineral and hybrid nanozymes (convergent evidence is growing today), and the impact craters/meteoritic minerals and solvent-controlled nucleotide stability would be an ideal concrete testbed.

In addition, the prebiotic synthesis of important organic molecules from dust-grain impacts might also contribute a lot to the OoL on Earth as in the process the magnetized turbulence drives dust drift/concentration and enhances nonthermal chemistry [68], which is conductive to the generation, enrichment, and chemical complexity of prebiotic organics on early Earth. Therefore, the above magnetized turbulence (with size/charge-selective dust dynamics that biases delivered feedstocks) and the bias reactivity of Fe-clay surfaces may work jointly and play key roles for the molecular (photo-)selection of the MN-zyme systems for the OoL on Earth.

## Conclusion

The OoL on Earth is a fundamental and long-standing scientific question. Although nowadays the emergence of the first biopolymers as well as their building blocks on the early Earth is considered an important moment in the OoL, how life began on the prebiotic Earth from a pile of prehistoric inert chemicals (gases) is still a puzzle to us. Over the past century, a variety of plausible OoL hypotheses have been proposed. However, each of these hypotheses can only explain relatively narrow parts of whole scenarios, no convincing “one-piece” hypothesis so far can unify them, to provide complete and credible scenarios of how original life can arise from a bunch of inert chemicals on the planet. In the nanozymes hypothesis, natural MN-zymes and their later upgraded organic/inorganic hybridized nanozymes played multiple key roles in the initial emergence of life molecules under primitive Earth conditions. Importantly, under the hypothesis framework, proteins, DNA, and RNA probably can form near-simultaneously (but need to note that although there are already initial experimental signs/clues [58–60], further experimental evidences under the topic are needed to prove this), as a result of the diversity of nanozymes and catalyses, and multiple physical and chemical key roles of the MN-zymes. It therefore may unify the existing hypotheses to provide whole scenarios of the OoL on Earth.

**Fig. 5.**
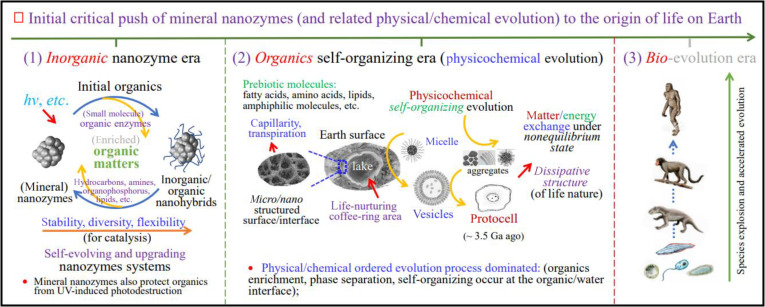
Schematic of initial critical roles of MN-zymes, and some key physical/chemical factors and main evolution stages, of the OoL on Earth.

Finally, Fig. 5 summarizes and depicts the simplified scenarios and a few key factors of the main evolution stages of the OoL on Earth, highlighting the initial critical push of MN-zymes, and subsequently emerged physical/chemical evolution driving forces, to the emergence of life on Earth. Note that, in the subsequent self-organizing era, the formation and self-organization of biomacromolecules and organic matters, especially the formation of vesicular-like or compartmentalized organic entities and later protocells, require the enrichment and concentration of substances and catalysts (MN-zymes). Therefore, it needs the sites or microenvironments on the Earth’s surface with alternating dry and wet conditions, where the MN-zymes work well to fulfill their diverse functions and the related physical/chemical ordered evolution processes dominated. The author hopes that the nanozymes hypothesis proposed herein will help clear up long-standing doubts about diverse hypotheses on the OoL on Earth and make substantial contributions to the final revelation of the OoL mystery.
